# Structural insights into selective small molecule activation of PKG1α

**DOI:** 10.1038/s42003-023-05095-4

**Published:** 2023-07-31

**Authors:** Essam Metwally, Victor Mak, Aileen Soriano, Matthias Zebisch, H. Leonardo Silvestre, Paul A. McEwan, Grigori Ermakov, Maribel Beaumont, Paul Tawa, John J. Barker, Rose Yen, Akash Patel, Yeon-Hee Lim, David Healy, Jennifer Hanisak, Alan C. Cheng, Tom Greshock, Thierry O. Fischmann

**Affiliations:** 1 Modeling and Informatics, MRL, Merck & Co., Inc., 213 E. Grand Avenue, South San Francisco, CA USA; 2 Discovery Chemistry, MRL, Merck & Co., Inc., 213 E. Grand Avenue, South San Francisco, CA USA; 3 Quantitative Biosciences, MRL, Merck & Co., Inc., Kenilworth, NJ USA; 4 Evotec (UK) Ltd, 114 Innovation Drive, Milton Park, Abingdon, Oxfordshire, OX14 4RZ UK; 5 Discovery Bioanalytics, MRL, Merck & Co., Inc., 213 E. Grand Avenue, South San Francisco, CA USA; 6 Discovery Biology, MRL, Merck & Co., Inc., Boston, MA USA; 7 Discovery Chemistry, MRL, Merck & Co., Inc., Kenilworth, NJ USA; 8 Protein and Structural Chemistry, MRL, Merck & Co., Inc., Kenilworth, NJ USA

**Keywords:** X-ray crystallography, Molecular modelling, Kinases, Target validation

## Abstract

cGMP-dependent protein kinase I-α (PKG1α) is a target for pulmonary arterial hypertension due to its role in the regulation of smooth muscle function. While most work has focused on regulation of cGMP turnover, we recently described several small molecule tool compounds which were capable of activating PKG1α via a cGMP independent pathway. Selected molecules were crystallized in the presence of PKG1α and were found to bind to an allosteric site proximal to the low-affinity nucleotide binding domain. These molecules act to displace the switch helix and cause activation of PKG1α representing a new mechanism for the activation and control of this critical therapeutic path. The described structures are vital to understanding the function and control of this key regulatory pathway.

## Abstract (web summary)

Structural characterization and modeling of cGMP‐dependent protein kinase I‐α (PKG1α) with a small molecule activator suggest the activation mechanism for the PKG1α homodimer.

## Introduction

Cyclic guanosine monophosphate (cGMP) dependent signaling has been implicated in the regulation of numerous pathways^1^. Its role in cardiovascular health cannot be understated. From promoting smooth muscle relaxation in response to nitric oxide^2,3^, to the prevention of platelet aggregation^4^, and even the attenuation of thrombin receptor protease-activated receptor-1 (PAR-1) via regulator of G-protein signaling-2 (RGS2)^5^, cGMP signaling acts to help maintain and modulate normal vascular function and blood pressure. Successful strategies for the regulation of pulmonary hypertension have focused on stimulation of soluble or particulate guanylate cyclase (sGC) with the first such therapeutic, Riociguat, approved for use in the United States^6,7^. Similar effects can be obtained through inhibition of cGMP degradation by phosphodiesterases (PDEs)^8–11^. However, targeting of sGC or PDE inhibition is ultimately non-specific as it acts to increase levels of cGMP which has roles in numerous downstream pathways, including signaling through cGMP-dependent protein kinase (PKG)^1,12^. By directly activating PKG1α, direct impact on cardioprotective mechanisms could be realized without the complication of the non-specific systemic effect of cGMP signaling^13^.

**Fig. 1:**
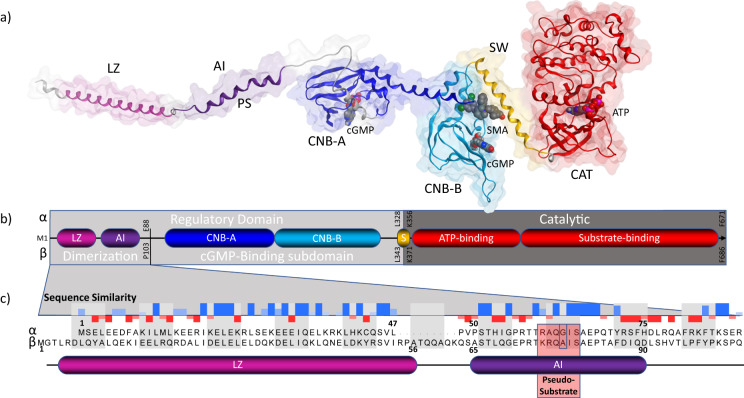
Domain architecture and organization of human PKG1. **a** PKG1α constructed monomer highlighting domains and subdomains as correspond to the schematic representation (**b**) of the domains with α visualized on top and β underneath. The domain residue boundaries for each isoform are indicated. The regulatory domain consists of a dimerization subdomain and cGMP-Binding subdomain. The dimerization subdomain consists of a Leucine Zipper (LZ) and auto-inhibitory region (AI) which plays a role in protein regulation via the Pseudo-Substrate (PS). While the cGMP-binding subdomain consists of a high (CNB-A) and low-affinity (CNB-B) cyclic nucleotide binding sites which directly bind cGMP. Progressive binding of cGMP to these sites ultimately causes release of the AI domain, and extension of the catalytic domain away from the regulatory domain allowing for ATP-mediated substrate catalysis. **c** The aligned sequences of α and β dimerization domains is visible. All differences in sequence between the two variants are restricted to this subdomain. A sequence similarity histogram is overlayed with high similarity indicated as bars above the line and low or no similarity as bars below the line.

PKG1 is found in two primary isoforms, PKG1α and PKG1β, that are the product of alternative splicing^14^. Cardiovascular expression of both isoforms is widespread and implicated in the downstream activation effects imparted by sGC-derived cGMP production^15^. While the domain organization is conserved between both isoforms (Fig. 1a), the heterogeneity is restricted to the dimerization domain, which contains both the leucine zipper and auto-inhibitory domains (Fig. 1b). Overall sequence identity is high (~90%), with differences in sequence being restricted to the first 89 and 104 residues for PKG1α and PKG1β (~28% pairwise identity), respectively, with 100% identity in the remainder of the sequences. The PKG1α isoform is more sensitive to cGMP concentrations than the 1β isoform suggesting that the difference in sensitivity is likely directly attributable to the dimerization domain which also contains an autoinhibitory (AI) subdomain^14,16,17^. Within the AI lies the pseudo-substrate sequence wherein the requisite S/T phosphorylation site has been replaced by G/A for α/β respectively^18,19^. This near uniform identity further underscores the disparity between structures reported for PKG1α and 1β (PDB: 3SHR and 4Z07, respectively)^20,21^.

cGMP mimetics have been the predominant mechanism leveraged to derive small molecule PKG1 activators and are susceptible to off-target activity^5^. Synthetic peptides have also been developed and were shown to reduce constriction of endothelium-denuded arteries^17^. These peptidic derivatives are thought to mimic, and thereby displace the switch helix and prevent PKG1α activity regulation^17,22^.

We have recently reported a series of piperidine-based small molecule PKG1α activators (SMA)^23–25^. These molecules were demonstrated to enhance the activity of PKG1α in a concentration-dependent manner. In cell-based assays, they were shown to facilitate phosphorylation of the known PKG1 substrate, vasodilator-stimulated phosphoprotein (VASP), and display anti-proliferative effects in human pulmonary arterial smooth muscle cells (hPASMC)^25^. Biochemical and biophysical studies along with initial modeling efforts, indicate an allosteric binding site proximal to the low-affinity cGMP-binding domain (CNB-B)^23^. In the full-length PKG1α, these compounds allosterically enhance the binding affinity of cGMP for the CNB-A site (positive binding cooperativity) and decrease the apparent affinity of cGMP for CNB-B site (allosteric competition). The piperidine series allosteric binding site was later supported by hydrogen-deuterium exchange (HDX) and ultimately confirmed by subsequent high-resolution X-ray co-crystals of the CNB-B domain with various SMAs bound^25^. In trying to rationalize the SMA mechanism of action, we attempted to crystalize larger fragments of PKG1α than had been previously described. Reported herein are high-resolution co-crystal structures for the truncated CNB-B coupled by the switch helix to the kinase/catalytic domain (CAT). These structures were obtained with various SMA, as well as cGMP with ATP to a 2.00 Å resolution. Reconstructions of PKG1α based on these crystals, in both activated and quiescent states, was also completed. This model provides detailed insight into the interaction cascade ultimately resulting in PKG1α-mediated phosphorylation of protein substrates.

## Results and discussion

### PKG1α small molecule activators bind allosterically to CNB-B domain

**Fig. 2:**
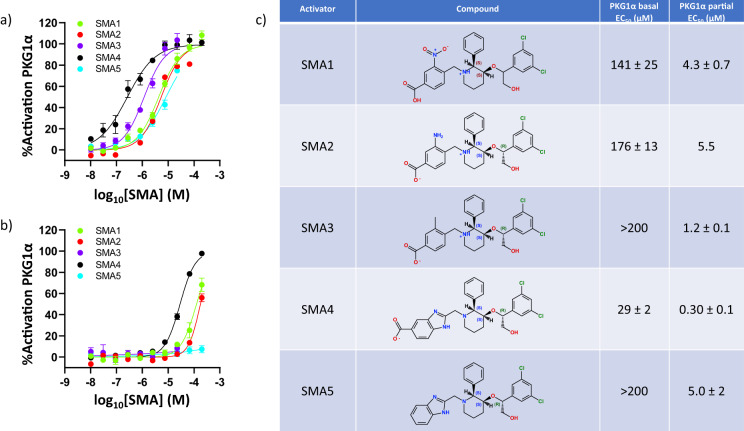
Enhancement of PKG1α activity at increasing concentrations of small molecule activators (SMAs). **a** Enhancement of activity of partially activated PKG1α (EC_20_ with 10 nM cGMP) and **b** enhancement of PKG1α from the basal state (no cGMP) at increasing concentrations of SMA1, SMA2, SMA3, SMA4, and SMA5 tracked using ADP-Glo kinase assay. **c** Table shows activation potency estimates (EC_50_) from non-linear fits of concentration-response curves from (**a**, **b**). EC_50_ values were the average of 3 to 6 independent trials (errors represented as ±SE).

A novel piperidine series of SMAs were previously identified through an ultra-high-throughput screen (uHTS) of a Merck & Co., Inc., Kenilworth, NJ, USA internal collection consisting of ~2.9 million compounds^24^. The uHTS hits and analogs from optimization efforts showed a concentration-dependent enhancement of activity of partially activated PKG1α (partial activation *at EC*_20_ in the presence of 10 nM cGMP, *V*_o_ ~ 0.13 μmol min^−1^ mg^−1^; for reference, *EC*_50_ ~ 30 nM cGMP corresponds to *V*_0_ ~ 0.25 μmol min^−1^ mg^−1^; please see Supplementary Fig. 1 for the full cGMP-induced PKG1α activation profile)^23–25^. Compounds from optimization efforts showed an ability to activate PKG1α independent of cGMP as exemplified by SMA1, SMA2 and SMA4 (Fig. 2)^25^. The enhancement of SMA activation potency (*EC*_50_) in the presence of cGMP (Fig. 2) is aligned with the observed increase in binding affinity of SMAs in the presence of increasing concentration of cGMP (Supplementary Fig. 2). These observed mutual modulation in binding affinities is attributed to the positive binding cooperativity observed between the piperidine series activators and cGMP bound to CNB-A^23^. The observed allosteric nature of the SMA binding site in full-length PKG1α is recapitulated in Supplementary Fig. 3 for SMA3 and SMA4 following a previously reported fluorescence polarization method tracking the modulation of cGMP affinity for CNB-A and CNB-B at increasing concentration of SMA^23^. The non-linear enhancement in the apparent cGMP affinity (*K*_d, app_) for CNB-A demonstrate the allosteric binding cooperativity between SMA and cGMP bound to CNB-A (Supplementary Fig. 3A). On the other hand, the non-linear decrease in apparent binding affinity of cGMP for CNB-B with increasing concentration of SMA recapitulates the previously reported negative binding cooperativity (allosteric competition) between SMA and cGMP bound to CNB-B (Supplementary Fig. 3B). This negative binding cooperativity was shown to be preserved in the isolated regulatory domain fragment (PKG1α_78–355_), supporting the localization of SMA binding site near CNB-B^23^. To better understand the binding characteristics of the activators, a docking model was built based on a previously reported PKG1α structure (PDB: 3SHR)^21^. Initial crystallization with the CNB-B domain and SMA1 confirmed binding at an allosteric site (Nest) proximal to the cGMP binding site^25^. Several SMA were tested for their capacity to co-crystalize with various constructs of PKG1α. While SMA1 was useful in the study of CNB-B, we were able to obtain co-crystals of the CNB-B/Switch helix/CAT constructs with SMA2, SMA3, and SMA5 for the described structural efforts (Fig. 2).

### Activators interfere with the switch helix

**Fig. 3:**
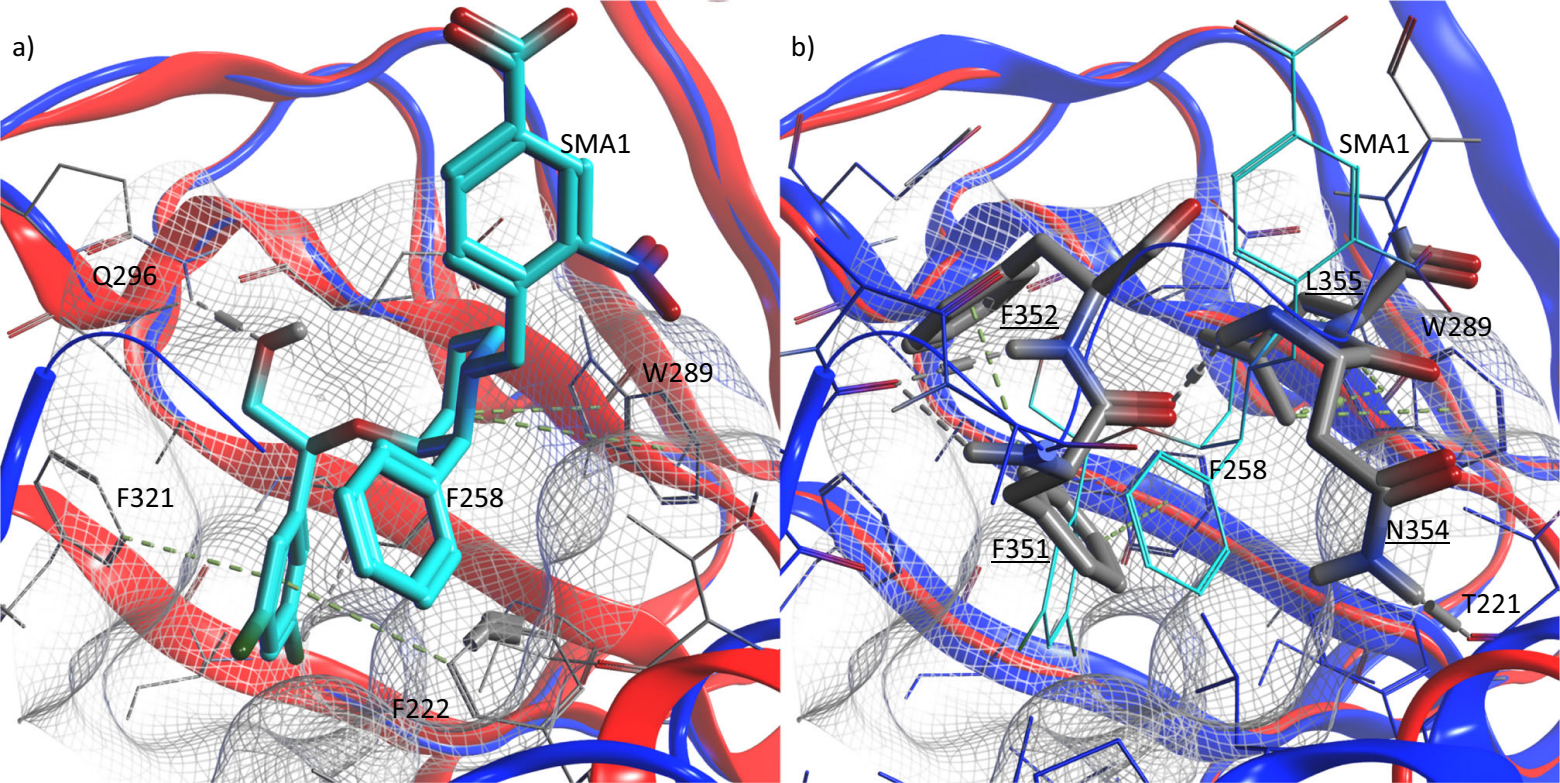
Comparison of the binding of SMA1 vs. trans-switch helix binding at the CNB-B nest. Overlay of 7SSR (red) and 3SHR (blue) with SMA1 (**a**), or the trans-chain switch helix (**b**) bound in the nest region of CNB-B. SMA1 is rendered in cyan sticks for reference. The ligand residues of the switch helix are comprised of F351, F352, N354, and L355 and are underlined to distinguish them from site residues. The nest is rendered with a mesh surface based on 7SSR. Residue-binding interactions between the site and the binding entity are labeled and indicated with dashed lines. Interactions with W289 and F258 are conserved, while SMA1 engages in additional interactions deeper withing the pocket including with F222 and F321. This is in addition to an interaction with Q296. Note how the dichlorophenyl moiety is positioned where F351 would normally have bound, but with increased binding interactions owing to its angle and depth. This increased level of binding interaction is consistent with its ability to displace the switch helix in partially activated PKG1α.

Initial results suggested that the SMA series bind within a described Nest region that is normally occupied by the switch helix. Like the previously described S-tides^17^, we hypothesized that the SMA were acting to displace the helix, forcing the structure into an open and activated state. To verify, several constructs of CNB-B were crystallized in the presence of SMA1 (PDB: 7SSB, Fig. 3a) to a resolution of 1.4 Å. This structure exhibited a backbone RMSD of 0.858 Å and an all atom RMSD of 1.449 Å to 3SHR (Fig. 3).

Although initial crystallization efforts reproduced the previously published crystal dimer (PDB: 3SHR)^21^, there remained questions as to how the missing domains (the leucine zipper (LZ) and CAT) would position themselves in relation to the crystallographic conformation. This inconsistency seemed to be more pertinent in the context of more recently reported structures for the closely related PKG1β isoform (PDB: 4Z07)^20^, as well as the crystallized catalytic domain (PDB: 6BDL)^26^. We hypothesized that the extended conformation may be due to the absence of the catalytic domain and, in its presence, PKG1α would more readily adopt a more compact homodimer with the switch helices serving in a self-regulatory role rather than influencing the dimeric counterpart as previously suggested by the ∆CAT-PKG1α structures, likely adopting an antiparallel dimeric arrangement^21,22^. This auto-regulatory function would seem to concur with observations that ∆LZ-PKGI monomers in solution exhibit almost identical activation profiles to their dimeric counterparts, suggesting that the regulatory function of the switch helix remains intact despite existing in a monomeric state^20,27,28^. Furthermore, previous studies with small-angle X-ray scattering (SAXS) suggested that in its fully activated state, PKG1α forms a highly asymmetric structure with a maximum linear dimension of about 165 Å in its basal state^29,30^. This increased length is characterized by movement away from the core toward the periphery.

### Crystallization of CNB-B and catalytic domain

**Fig. 4:**
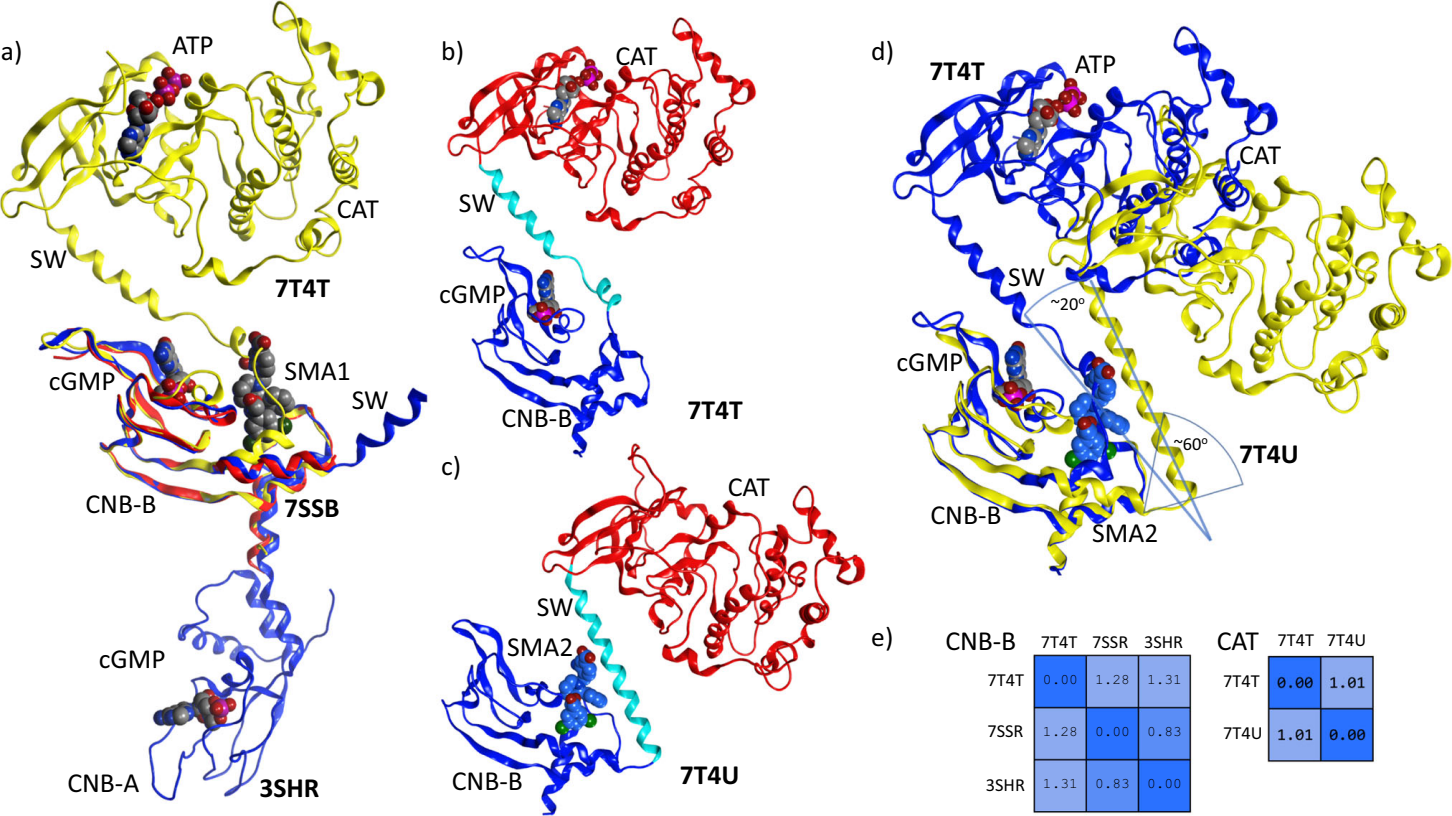
Crystallographic overlay and comparison of the cGMP binding subdomain with CNB-B, and the CNB-B/SW/CAT. **a** Overlay of 3SHR (blue) with 7T4T (yellow) and 7SSR (red) on the CNB-B domain. Note the dramatic shift in the switch helix (SW) position in the presence of the catalytic domain (CAT). **b** Structure of the C-terminal domains of PKG1α 7T4T (CNB-B/SW/CAT + ATP + cGMP), **c** 7T4U + SMA2. CNB-B is colored blue, SW is colored cyan, and CAT is colored red. **d** Overlay of (**b**) and (**c**) with 7T4U in yellow and 7T4T in blue. Note the residues leading into SW rotate out about 60° before turning back. The SW with SMA2 bound, rotates away from CNB-B by about 20° vs. the orientation found in the cGMP-bound state. **e** RMSD-Matrices of both the CNB-B and CAT domains demonstrate that each acts as a rigid-body with changes in orientation or presentation relegated to the SW.

**Fig. 5:**
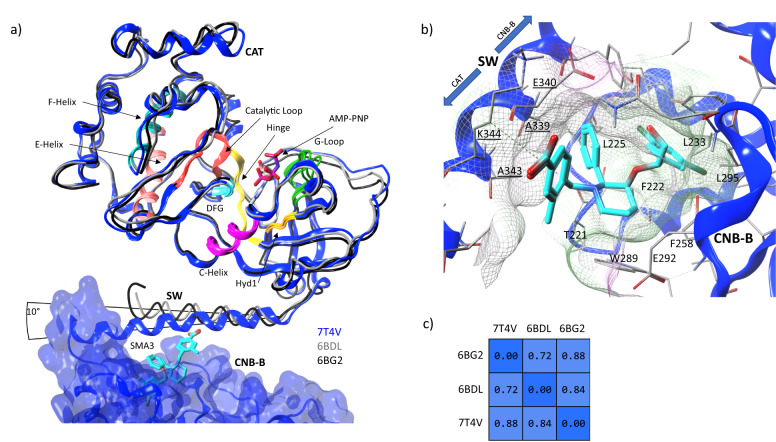
Crystallographic overlay of the catalytic domain (CAT) with the CNB-B/SW/CAT crystal and the impact of SW on the CNB-B nest. **a** Catalytic domain of PDB: 7T4V in blue ribbons is overlaid with the apo structure in gray (PDB: 6BDL) and ATP-bound in black (PDB: 6BG2) both as tubes. The kinase regions have been annotated to provide orientation and context. The observed all-atom RMSD for the domain is extremely low with much of the deviation occurring in the regulatory switch helix which exhibits a change in its exit angle of about 10°. AMP-PNP of 6BG2 is visualized in the ATP pocket. SMA3 is displayed in the nest region of CNB-B. The catalytic domain itself behaves largely as a rigid body as can be observed by the traces and by the pairwise RMSD matrix (**c**) of residues 360–671. When examining the SMA3 position within the nest domain (**b**) of CNB-B, the SW helix residues (underlined) contribute to the binding site previously observed by adding an additional surface for interactions. A339 from the helix works with L225 and T221 to complete a hydrophobic pocket occupying the phenyl substituent while the methylbenzoic acid moiety interacts with A343 and K344. The mesh surface is colored from lipophilic (green) to hydrophilic (purple).

To better understand the structure and the impact of the uncrystallized domains, the CNB-B domain, switch helix, and complete catalytic/kinase domain were crystallized in the presence of ATP and cGMP (PDB: 7T4T) to 2.0 Å resolution (Fig. 4). When aligning and superposing the catalytic domain with AMP-PNP bound (PDB: 6BG2), the all-atom RMSD was found to be under 1 Å (Fig. 5). While we were principally interested in the impact of cGMP binding, ATP was found to stabilize the construct, facilitated crystallization, and improved the final resolution. The combined domains were also crystallized in the presence of three SMAs (PDB: 7T4U, 7T4V, 7T4W for SMA2, SMA3, and SMA5 respectively). Attempts to crystallize the construct in a basal or apo form were unsuccessful. As can be observed (Fig. 4c), SMA2 has a marked impact on the protein conformation, forcing the regulatory helix to open and rotate out by as much as 30 degrees. The catalytic domain behaves as a rigid body (Fig. 5) with no change in local conformation. When the regulatory loop is excluded, the domain exhibits an all-atom RMSD of only 0.94 and 1.18 Å to previously published apo- and AMP-PNP-bound structures (PDB: 6BDL and 6BG2 respectively)^26^. The key difference between structures lies in the regulatory helix which rotates about 20° away from CNB-B in the presence of the SMA (Fig. 4d). This results in the catalytic domain shifting by about 30 Å from the cGMP and ATP bound state. Identical shifts were observed with all 3 SMA (PDB: 7T4U, 7T4V, 7T4W). Statistics for the crystallographic data are summarized in Table 1.

**Table 1:** X-ray data and refinement statistics for PKG1 CNB-B and CNB-B-SW-CAT domain constructs.

Data collection	7T4T	7T4U	7T4V	7T4W	7SSB
Protein	CNB-B-SW-CAT	CNB-B-SW-CAT	CNB-B-SW-CAT	CNB-B-SW-CAT	CNB-B
Ligand(s)	cGMP, ATP	SMA2	SMA3	SMA5	SMA1
Wavelength (Å)	0.9686	0.91587	0.9686	0.91587	0.9792
Space group	P 1 2_1_ 1	P 2_1_ 2_1_ 2_1_	P 2_1_ 2_1_ 2_1_	P 2_1_ 2_1_ 2_1_	C 2 2 2_1_
Cell dimensions					
a, b, c (Å)	73.120, 96.750, 81.130	93.463, 102.709, 103.843	92.232, 103.690, 104.310	93.000, 102.621, 104.028	56.394, 80.656, 63.222
α, β, γ (°)	90, 93.58, 90	90, 90, 90	90, 93.58, 90	90, 90, 90	90, 90, 90
Resolution (Å)	80.974–2.073 (2.07–2.26)	73.02–2.00 (2.17–2.01)	73.54–2.29 (2.60–2.29)	93.00–2.24 (2.58–2.24)	63.22–1.40 (1.42–1.40)
*R*_merge_	0.061 (0.941)	0.170 (1.278)	0.221 (0.941)	0.0145 (0.447)	0.160 (0.345)
*I*/*σ*_I_	11.6 (1.5)	8.8 (1.5)	6.5 (1.5)	8.0 (1.8)	4.0 (1.5)
Completeness (%)	94.0 (67.7)	93.9 (75.4)	92.9 (68.4)	90.6 (52.2)	97.0 (97.4)
Redundancy	3.4 (3.5)	6.2 (6.2)	5.8 (4.2)	5.0 (1.6)	2.7 (2.0)
Refinement					
Resolution (Å)	29.50–2.08	39.36–1.99	34.16–2.28	73.06–2.23	28.20–1.40
Number of reflections	46,106	45,638	22,609	28,165	27,741
*R*_work_/*R*_free_^a^	0.188/0.221	0.214/0.260	0.194/0.256	0.209/0.268	0.210/0.225
Number of atoms					
Proteins	7240	7402	7423	7446	966
Ligand	143	92	94	92	37
Water	250	307	107	141	156
B factors					
Overall (Å^2^)	45.22	34.63	38.79	30.54	8.70
Rmsd					
Bond lengths (Å)	0.010	0.010	0.010	0.010	0.010
Bond angles (°)	1.00	1.03		1.04	1.05

Parenthetical values are for the highest-resolution shell.

^a^5% of observed intensities excluded for calculation of *R*_free_.

In the ATP and cGMP-bound form of the current CNB-B/SW/CAT construct, crystals grow in a different space group (P2_1_ a = 73.1 Å b = 96.7 Å c = 81.1 Å *β* = 93.6°) that is different from that obtained (consistently) from the same construct with any of our SMA ligands (P2_1_2_1_2_1_ a = 93.2 Å b = 103.2 Å c = 104.1 Å). Two copies of the molecule were observed per asymmetric unit but, as with previous structures with the same construct, the crystal packing was consistent with a monomeric oligomerization state in solution. This was confirmed via visual inspection and PISA analysis. Chain B displayed slightly more ordering than chain A but, in all cases, there was well-defined electron density for ATP and cGMP.

In examination of the switch helix, the N-terminal half of the helix packed against CNB-B, contributing to the cGMP binding surface. While this appears to be consistent with the switch helix occupying the allosteric activator binding site, an apo-structure could not be crystalized for confirmation of the packing mode of the helix against the nest binding site. With respect to the C-terminal end of the helix, contacts between a small region of the N-terminal lobe of the catalytic/kinase domain and the last two turns of the switch helix can be observed (Fig. 4).

Attempts to soak activator with crystals reproducing the published regulatory domain structure (PDB: 3SHR) failed to produce co-crystals. This appears to be consistent with the switch helix contributing to a closed state of the Nest. Or put another way, competitive binding for the Nest between SMA and SW yielded more stable crystals with the helix bound. However, a co-crystal with SMA1 (PDB: 7SSB, Fig. 3a) and a construct encompassing only CNB-B was obtained^25^. The binding of SMA1 appears to be consistent with the pocket previously occupied by the switch helix in 3SHR (Fig. 3b). In the current CNB-B/SW/CAT construct, electron density observed for SMA3 allowed for unambiguous determination of binding pose. In particular, chirality of the 3 chiral centers was clearly defined. Furthermore, SMA2 (Fig. 4), SMA3, and SMA5 were found to bind unambiguously to the Nest found on CNB-B (PDB: 7T4U, 7T4V, 7T4W).

As noted, the switch helix of the current crystal differs markedly from that previously described (PDB: 3SHR). Notably, the C-terminal end of the switch helix folds back and packs against CNB-B as opposed to pointing away from its monomer (Fig. 4a). While there are many possibilities that could explain this discrepancy, the simplest is the presence of the kinase domain providing the necessary contacts and structure to maintain the switch helix in a packed state, locking it into place between CNB-B and CAT. In the absence of the catalytic domain, stability is found by packing the switch helix against the adjacent monomer Nest of CNB-B yielding the previously observed extended structure (PDB: 3SHR) (Fig. 3b).

It is clear that the CAT domains do not contribute to the binding domains of CNB-B, whether the low-affinity cyclic nucleotide binding site, or the Nest. CAT appears to be well isolated and only becomes active when PKG1α is in an active state elicited through cGMP binding or the presence of an activator. The kinase domain packs against the C-terminal region of the switch helix, and, most notably the kinase is phosphorylated at Thr517 with Glu409 in the correct position for catalysis.

In contrast, the switch helix does indeed contribute to the CNB-B SMA binding site as can be seen from 7T4V (Fig. 5). The switch helix is observed to cross over SMA3. In the close-up (Fig. 5b), the phenyl substituent off the piperidinyl 2-position is enclosed in a hydrophobic pocket formed by L225 and T221 which is completed by the Ala339 of the SW. The methylbenzoic acid moiety on 1-nitrogen packs along the peptide bond between Ala343 and Lys344; interactions are mostly hydrophobic or involve π–π stacking.

### SMA selectivity for PKG1α

The SMA have previously been demonstrated to preferentially activate PKG1α over the PKG1β isoform^25^. In vivo, this selectivity would be further enhanced by the higher affinity of the α-isoform for cGMP over the β-splice variant^19,31^. It has been previously shown that there is a 6–10 fold increase in the K_D_ of cGMP for PKG1α over PKG1β^32,33^. SMA3 required 6.5x, while SMA1 and SMA2 a 12x increase in concentration to elicit the same level of activation on PKG1β in the presence of 100 nM cGMP vs. the 10 nM used for PKG1α^25^. It is important to note that the reported activities for both PKG1α and PKG1β were determined with full-length protein. This preferential activation of α was also observed by Moon et al. and their synthetic peptides^17^. Given that the differences between the two isoforms is isolated to the dimerization domain (Fig. 1), this domain must play a direct role in the regulation of the system as a whole. A multifactor regulation system involving the leucine zipper, auto-inhibitory domain, and the pseudo-substrate all appear to contribute to this differential cGMP sensitivity and potentially may have a similar effect on the binding profiles of the SMA^33,34^. Aside from acting to dimerize the monomers, the LZ does not appear to play a direct role in the regulation of PKG1. We hypothesize that the LZ acts to dimerize the system and maintain the AI sequences in proximity to the CAT domains of each monomer. It is likely that the increased sensitivity of α over β can be attributed to being 14 residues shorter in the dimerization domain (Fig. 1c). This reduced chain length potentially results in a higher probability of disassociation, either spontaneous or in response to binding at CNB-A of cGMP. Whatever the mechanism, it does suggest that preferential activation of PKG1α within a viable therapeutic window is possible.

### Protein structure

While crystal packing may influence quaternary structure, to assess the impact of the environments on each of the crystals, we directly compared the monomers in the crystal asymmetric units by independently superposing on either CNB-B or CAT (Fig. 5a). This revealed no marked differences within the individual moieties due to either the presence of activator or cGMP. However, perturbations were observed in the orientation of the switch helix relative to CAT and CNB-B and were the only appreciable changes observed due to ligand binding (Fig. 4). The presence of ATP did slightly alter the elbow angles between N- and C- terminal lobes which is consistent with other kinases. The helix rotates between 20° and 30° away from CNB-B with the catalytic domain behaving as a rigid body at the end of the helix (Fig. 4c). While the bulk of CNB-B remains relatively constant in conformation, as the sequence begins to transition from CNB-B to the helix, there are structural rearrangements which directly contribute to the rotation, translation, and relative orientation of the helix to CNB-B. To underscore the scale of motion, the relative position of the Cα of Tyr336 is observed to deviate from its initial position by ~19 Å.

Overall, the data suggest that both cGMP and the SMA induce structural rearrangements where the switch helix is forced to rotate and translate out, causing the helix to pack against CNB-B and ligand as opposed to CNB-B alone. Despite these similarities, the specifics of the molecular interactions differ markedly as does the overall shape of the enzyme upon ligand binding (Fig. 4d).

### Hydrogen-deuterium exchange (HDX)

**Fig. 6:**
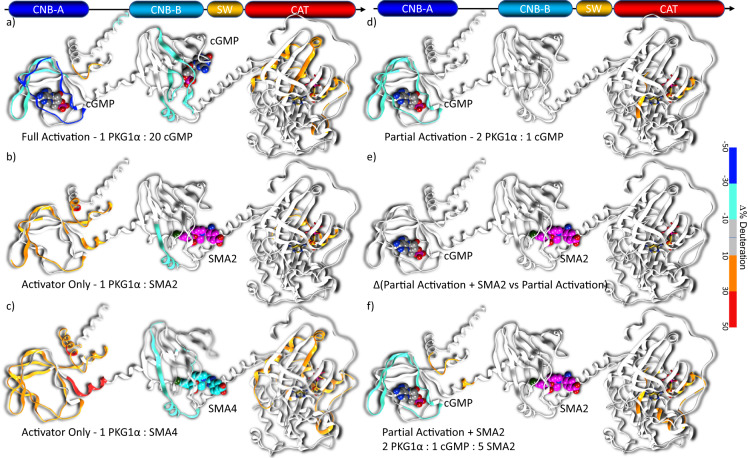
Overlay of hydrogen deuterium exchange data on the protein ribbon of PKG1α homology model. Ligands are displayed in space-fill with cGMP colored by element, and two activators colored with magenta (SMA2), or cyan (SMA4) carbons. ATP is rendered in gold sticks only for reference. Ribbons for regions with decreased proton exchange are blue/cyan, while higher exchange regions are colored in red/orange. All traces indicate the change in deuteration with respect to PKG1α alone. Ligands have been overlaid to indicate the experimental conditions. In all scenarios, binding of ligand causes a corresponding decrease in deuteration with proportional increases in deuteration observed at the ATP-binding site indicating increased accessibility of the site in response to activation. **a** 1:20 PKG1α to cGMP, cGMP binds to both high and low-affinity CNB domains. **b**, **c** SMA2 and SMA4 bound to the allosteric activation site. **d** 2:1 PKG1α to cGMP, **e**, **f** 2:1:5 PKG1α:cGMP:SMA2 with respect to (**d**) and (**e**), respectively. While (**e**) shows no appreciable proton exchange in response to SMA2 over the partially activated state (**d**), there is a subtle yet appreciable increased in deuteration which can be observed by comparing to the basal state (**f**).

Deuterium exchange mass spectrometry (HDX-MS) experiments with full-length PKG1α have revealed that cGMP-mediated activation results in a more solvent exposed autoinhibitory domain and hinge region, as well as a disclosure of residues within the substrate domain^35^. HDX-MS experiments were performed to elucidate the mode of activation by the small molecules such as SMA2 and SMA4 (Fig. 2) that demonstrate potency in partial and basal states of PKG1α (Fig. 6). The change in deuteration when compared to the inactive state clearly demonstrates an increase in the accessibility of the ATP-binding domain when PKG1α is in an activated conformation. This accessibility is clearly visible in the high cGMP condition (Fig. 6a), and with both SMA2 and SMA4 to varying degrees, with one activator yielding deuteration changes comparable to the partially activated state (Fig. 6d) and the other comparable to the fully activated state (Fig. 6a). Furthermore, the observed decreases in deuteration were consistent with molecules binding at the indicated sites whether cGMP or activator elicited decreases in deuteration. HDX offers compelling support for allosteric binding of activators at a site proximal to CNB-B in the full-length protein. Interestingly, the high-affinity CNB-A site remains unoccupied in the presence of activator and demonstrates increased levels of deuteration (Fig. 6b, c) offering further confirmation that the observed activation caused by small molecule binding is due to action at a site distinct from that of cGMP.

### In silico structural models: apo-CNB-B-kinase model

**Fig. 7:**
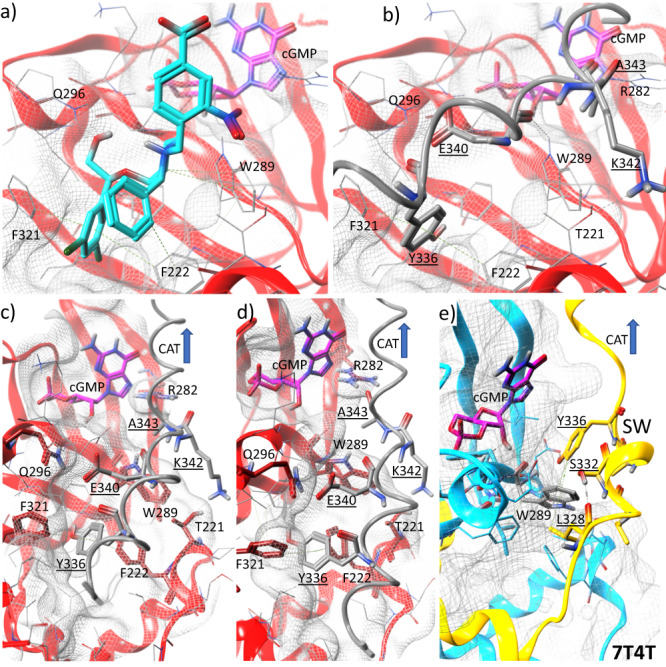
In silico model templated on 7SSB for CNB-B and 7T4V for the switch helix (SW) to form the apo state of CNB-B with the switch helix packed against the domain. **a** SMA1 (cyan) and cGMP (magenta) are rendered for reference in the Nest and cyclic nucleotide binding sites respectively. Surface is rendered in wire. **b** The switch helix is rendered in gray tube and line to facilitate visualization. Key residues of the switch helix labeled and underlined. Note the tight fit of Tyr336 within the same hydrophobic cleft as the dichlorophenyl group of SMA1. H-arene interactions occur with both Phe222 and Phe321. The helix appears to be held in place with strong salt-bridges with interactions occurring between Glu340, and Gln296 as well as maintaining the helix through a salt bridge between Lys342 and T221. An additional backbone interaction with W289 is also observed in both sets of interactions. **c**, **d** Alternate views of packed helix against CNB-B, note how the cGMP binding site is not restricted. **e** In the cGMP/ATP bound structure of 7T4T, with CNB-B in cyan and SW in gold, interactions with the partially disordered switch reveals that that Leu328, Ser332, and Tyr336 act to strongly chelate Trp289 which seems to be the focus of the helix interactions with the nest in the cGMP activated structure.

Based on the observation that CNB-B and CAT domains behave as rigid bodies tethered by the switch helix, the activator was removed from the structure and the CAT/SW were rotated and translated toward the nest domain. This movement brought Tyr336 into the region of the Nest and placed the helix in complete coordination with the previously occupied Nest and partially occluding the low-affinity cyclic nucleotide binding site of CNB-B. The minimized structure demonstrated an ideal overlay of Tyr336 and Glu340 directly replacing and replicating the interactions observed with the SMA1 (Fig. 7). Based on molecular mechanics forcefield calculations, identical H-Arene interactions between the phenyl moiety of either the activator or the helix Tyr336 and both Phe222 and Phe321 are observed. In the case of Tyr336, these H-Arene interactions are on the order of ~0.6 kcal/mol weaker vs. those found in the activator. This is likely due to the increased ability to penetrate deeper into the cleft as the small molecule is not restricted through tethering to the helix, yielding slightly improved stacking interactions (Figs. 3a and 7a). Interactions with Trp289 are also maintained, however, a weak interaction between the carbonyl backbone of Glu340 acts in place of an almost 1 kcal H-arene interaction from the central ring to that of the histidine. Additional kcal/mol is observed in the helix interaction forming a salt bridge with Gln296. This strong interaction is the likely reason that many of the activators required high concentrations to elicit PKG1α activation^25^. The close correspondence between activator and helix interactions with CNB-B lends strong support to the hypothesis that PKG1α regulation is directly mediated through binding of the switch helix to the Nest. Furthermore, the activity of the described activators, whether S-tides^17^ or SMA^25^, likely stems directly from the capability of these compounds to mimic and displace the switch helix from the Nest moving the CAT domain into an unprotected and open state.

### In silico structural models: PKG1α-activated complex

**Fig. 8:**
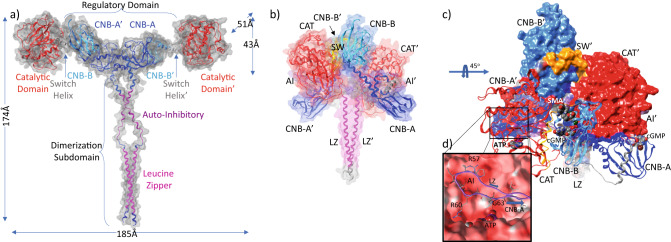
Domain architecture and organization of human PKG1α in activated and auto-inhibited apo states. Reconstructed PKGIα homodimer based on crystal structures of all domains. Domains are colored per schematic found in Fig. 1. LZ (magenta), AI (purple), CNB-A (blue), CNB-B (cyan), SW (gold), CAT (red). Overlays of domain demonstrate all-atom RMSD on the order of <1.0 Å. The models were templated on Leucine Zipper (LZ) (PDB: 4R4L), Regulatory Domain (PDB: 3SHR), CNB-B/SW/CAT (PDB: 7T4V). In the activated state (**a**), the link between LZ and the regulatory domain was modeled into place and is the only domain without a crystallographic basis. The cyclic nucleotide-binding sites beginning with the high-affinity site (CNB-A) and low-affinity (CNB-B) sequentially activate through binding of cGMP to eventually allow for activation of the catalytic domain through movement and exposure of the catalytic-domain. SMAs bind adjacent to the low-affinity CNB-B cGMP binding site. **b** Notable in the auto-inhibited apo state is the compactness of the structure, the CAT domain sits atop the regulatory domain (CNB-A/CNB-B) of the paired chain but is inhibited by the AI of its own chain. In (**c**), the system is rotated toward the viewer by 45° to help visualize the auto-inhibition. The first chain is rendered in ribbons, with the second chain with a molecular surface colored by domain. From this perspective the single chain organization is more readily apparent. Ligands have been placed in their binding pockets for reference rendered and in spacefill. Note the positioning of the SMA in the nest region of CNB-B cGMP binding site. The SMA overlaps with the N-terminal of SW helix in this state. Both CNB-B and CNB-A binding sites are solvent accessible in this state. **d** Zoomed in view of the AI domain bound in the catalytic site. Note the putative phosphorylation site of the substrate is currently occupied by G63 of the pseudo-substrate resulting in the currently auto-inhibited state.

To complete the model, an in silico reconstruction was derived from an in-house structure which replicated the published regulatory domain (PDB: 3SHR) and our low-affinity nucleotide binding domain/catalytic domain structure (PDB: 7T4V) (Fig. 4). The LZ or dimerization domain was templated to a previously published structure (PDB: 4R4L) with the missing auto-inhibitory domains and coupling modeled in place. The partially activated 3SHR state and its close concordance with our internal structures as well as the CNB-B of our CNB-B/SW/CAT constructs yielded a combined regulatory domain and catalytic structure that required minimal alterations to the model. We selected a partially activated state as it was most congruous with insertion of our SMA or cGMP at the CNB-B binding sites. The resulting model closely agrees with reported experimental measurements, yielding a maximum measured interatomic distance of 185 Å and radius of gyration (Rgyr) of ~62 Å (Fig. 8a). While the distance is well within the reported 25–30% increase in size from basal state, Rgyr is slightly above the upper threshold of 60 Å^29^. This value was determined at a 20:1 cGMP PKG-dimer molar ratio, and is predicated on saturation by cGMP. As can be observed (Fig. 4d), occupancy of the CNB-B nest causes an increase in helix angle by ~20° and further extension of CAT away from the central core of the dimer. An increase in Rgyr of ~2 Å would be in-line with these observations.

### In silico structural models: PKG1α auto-inhibited complex

The recently published auto-inhibited structure of PKG1β (PDB: 7LV3) exhibits trans auto-inhibition via binding of the pseudo-substrate to the CAT domain^19^. Individual domains in this auto-inhibited state are extremely similar in structure, exhibiting sub-1Å RMSD. Despite the crystallographic trans-inhibition, evidence is suggestive that cis-inhibition is the dominant form of regulation^19^. To investigate this further structurally, we generated the monomer using AlphaFold^36^. The generated monomer (Supplementary Fig. 4a) was superposed on the CAT domain of 7LV3. Initial superposition failed to properly align the structures likely due to assignment of the CAT domain with CNB-A/B on the disconnected density. As per the authors of 7LV3^19^, it is possible to interpret the missing density as being contiguous with the co-located domains. When this was done, superposition was largely consistent with minor deviations in position owing to the alpha-helical connections between domains (Supplementary Fig. 4b). However, a high degree of concurrence can be observed for the individual domains. Additionally, the pseudo-substrate is bound within the catalytic site with the remainder of the LZ extended arbitrarily into solution. Since monomeric PKG1 exhibits self-regulation^20^, it is likely that this is a reasonable conformation for the complete auto-inhibited PKG1α monomer (Supplementary Fig. 4a).

We then leveraged AlphaFold-Multimer^37^ to investigate whether it would similarly predict cis-inhibition, or would recapitulate the published trans-inhibited structure. Unsurprisingly, the AlphaFold generated structure adopted trans-inhibition but was unable to draw conclusions about the dimeric nature of the LZ helices (Supplementary Fig. 4c). The structure was likely influenced by the existence of 7LV3 in its training data. Despite the issues, this became a start-point for the construction of the apo-autoinhibited model.

As with the reconstruction for the activated PKG1α, we used a LZ template (PDB: 4R4L) to build a complete model. LZ replaced the modeled zipper helices and was placed along the central axis to minimize the distance from each member of the dimer in both the trans and cis AlphaFold models. The system was minimized in MOE treating each lobe as a rigid body. This meant that for the trans-model a lobe consisted of a chain’s CNB-A, CNB-B, with the opposite chain CAT. While for the cis-model, CNB-A, CNB-B, and CAT were derived from the same chain. The switch helix as well as the auto-inhibitory region between the LZ and the PS were allowed to relax freely. The LZ was also treated as a rigid body.

While both the cis and trans dimers are computationally accessible, the “trans” dimer helix is consistent with the structures described herein (PDB: 7T4T, 7T4U, 7T4V, and 7T4W) with the CNB-B and CAT domains joined by the SW α-helix. This conformation has the added feature that in the inhibited state CNB-B SMA binding site remains occluded by the SW helix. Based on Sharma et al.^19^, PKG1β and likely PKG1α, appear to be auto-inhibited in cis. In the reconstruction of the model, the position of the LZ and the AI facilitates a “domain-swap” of the AI from the opposite chain. In this way, cis-inhibition is correctly encapsulated. This resulted in the final model (Fig. 8b) wherein the CNB-A and CNB-B flank CAT of the opposite-chain. The cis-AI region is positioned to facilitate auto-inhibition (Fig. 8c, d). Additionally, the positions of CNB-B, SW, and CAT are consistent with the crystal structures published to date.

### Proposed mechanism of activation

**Fig. 9:**
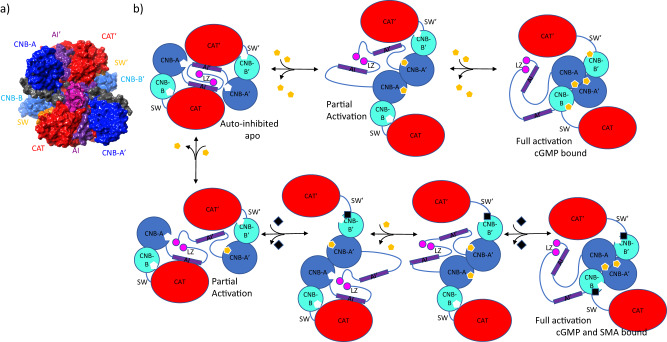
Proposed mechanistic pathway for PKG1 activation and deactivation using cGMP and/or small molecule activators. **a** Auto-inhibited apo-PKG1α as viewed from along the axis of the LZ helices (magenta), leading into the AI (purple) sequences, these loop around and auto-inhibit the CAT domain of the same chain. **b** In the rendered cartoon of the proposed mechanistic path, the domain organization of the dimer is much more apparent with the LZ serving to lock the dimers in place while appropriately presenting the AI domains of the cis-chain to each CAT domain. While basal activity could be mediated by transient disassociation of the AI from the CAT domain (not pictured), the apo-autoinhibited state serves as a start point for activity. This proceeds to the partially activated state where through binding of one or two cGMP (gold hexagon) to CNB-A domains elicits disassociation of the AI domain from CAT permitting the system to initiate extension away from the central axis of the dimer. This action draws CNB-A of each chain into close-proximity, further extending CNB-B and the CAT domain away from the core. Subsequent binding of cGMP to CNB-B displaces the SW helix further displacing and extending CAT into a completely open and accessible state. An almost identical process occurs in the presence of the SMA (black square) with the exception being that the second cGMP can be replaced by the SMA binding to the nest domain which also elicits full activation.

The construction of the complete dimer in both a compact apo-autoinhibited state and a fully extended activated state has simplified our understanding of the mechanics of regulation, activation, reorganization, and catalysis (Fig. 9). In the absence of cGMP, a compact auto-inhibited state exists whereby the AI sequence binds to the catalytic domain of a monomer. This is true in monomeric or dimeric states. It is auto-disassociation of the PS and AI domains which results in basal kinase activity that may be present in the absence of cGMP. Hydrogen/deuterium exchange experiments have indicated that the AI domain in PKG1β undergo increasing levels of deuteration with time in the absence of cGMP^38^, this is consistent with transient release and reassociation. There is no evidence to suggest that a similar mechanism is not in play with the α-isoform.

We hypothesize that the dramatic increase in activity of the SMA in the partially activated state is likely due to this auto-inhibition. When the AI domain is bound, the compact structure prevents movement of CAT away from the central core of PKG1. As can be seen in the in silico apo model, the switch helix crosses directly through the SMA placed for reference (Fig. 8c). This would cause the SW to remain bound with the nest site preventing binding of the SMA (Fig. 7b–d). During auto-disassociation or partial activation, the AI tether no longer holds CAT in place allowing for opening of the nest site at CNB-B (Fig. 4c, d).

Basal activity aside, the regulated pathway is cGMP dependent and normally would require a cGMP to activate. The proposed structure, consistent with that recently described for PKG1β^19^, has both CNB-A and CNB-B cGMP sites exposed to solvent. In theory, either site could bind cGMP first, but binding at CNB-B is unlikely to elicit activation particularly if the AI domains are engaged. It is binding at CNB-A which elicits disassociation of AI from CAT, beginning a counter-clockwise rotation of each cGMP-bound monomer of about 30° in plane. This brings CNB-A and CNB-A’ in to form the core of the diamond shape observed in 4Z07^20^. In this partially extended state, PKG1 is catalytically active though at reduced rate (Supplementary Fig. 1). On binding of the second cGMP the switch helix extends out from the CNB-B nest region, destabilizing the switch helix and (Fig. 4b) and encouraging alternate interactions near the nest of CNB-B (Fig. 7e), further extending the structure away from the core of PKG1. In contrast, binding of the SMA within the nest causes stabilization of the helix as can be observed when comparing the overlaid SW in Fig. 4d. This stabilization is due to the direct interactions observed between the SMA and SW (Fig. 5b). The CNB-B nest is capped by the SW, complementing the SMA’s features by enhancing the hydrophobic pocket and providing interacting residues for the solvent exposed regions (Fig. 5b). At this maximal extension, the catalytic binding site is fully exposed with limited accessibility and possibility for engagement or binding of the AI, provided cGMP or SMA are bound.

## Conclusion

The structures of CNB-B, switch helix, and catalytic subunit together provide an atomic view of how these domains interact in an activated state. The clear visualization of the binding region for the SMAs provides precise information on the interactions and mechanism involved in moving from a closed/inactive state to an open and active state. This activation is greater than the activation afforded by sub-maximal concentrations of cGMP alone. But more importantly, the combination of the CNB domains with the catalytic domain are suggestive of a novel activated-state structure that has not been described for PKG1α previously. Additional modeling and integration of these structures with the recently published auto-inhibited PKG1β monomer have provided the necessary insight to suggest a viable model of the auto-inhibited PKG1α dimer that remains consistent with our experimental knowledge of this protein kinase to date. The proposed model of activation would be applicable to both PKG1α and PKG1β. All published structures for both isoforms have been of the monomeric protein, though dimers have been naturally found for the activated regulatory domain. We look forward to future structural validation of our suggested models of the protein dimer to complete our understanding of the PKG1α/β system.

## Methods

### Sequence number

All sequence IDs are given with respect to UniProtKB for cGMP-dependent protein kinase 1 isoform Alpha, ID:Q13976-1 (KGP1_HUMAN)^39^.

### Molecular forcefield

Structures were prepared and minimized with the AMBER14:EHT forcefield^40,41^.

### Docking

Molecules were docked using the docking application in MOE 2019.0102^42^ using the triangle matcher placement algorithm with up to 100 poses retained for placement and 50 poses for a final refinement.

### Homology model generation

Homology models were initially created using 3SHR as a template using the Homology Modeler application in MOE 2019.0102^42^. These models were further refined and enhanced through reconstruction, overlay, and optimization with co-crystal structures in MOE 2019.0102. The dimerization domain was manually modeled and linked to the leucine zipper and regulatory domain.

### AlphaFold2

AlphaFold v2.2.4 was deployed and used to generate both a monomeric and dimeric structure^36,37^.

### PKG1α ADP-Glo kinase assay

SMAs were assessed in their ability to activate full-length PKG1α (human PKG1α_2–671_) kinase activity (from the basal and partially activated state) closely following previously published procedure^23–25^. Briefly, a luminescence-based detection method (ADP-Glo, Promega) was used to track kinase activity. Enzyme and substrate working solutions were prepared using the following assay buffer conditions: 50 mM Hepes, 10 mM MgCl_2_, 150 mM NaCl, 1 mM EDTA, 0.1 mg/ml bovine serum albumin (BSA), 5 mM β-mercaptoethanol (BME), 0.01% Triton X-100, pH 7.3 (BSA, BME, and Triton X-100 were added fresh to stock buffer solution on the day of experiment). Test compounds in DMSO solution were acoustically added (200 nl) to a 384-well white microplate followed by addition of an 8 μl mixture of PKG1α and peptide substrate, Glasstide (Anaspec) in the presence or absence of 10 nM cGMP. The compound and enzyme mixture was preincubated for 30 min at room temperature (RT) prior to addition of 2 μl of ATP to start the reaction (final assay conditions: 5 nM PKG1α, 150 μM Glasstide, 1 mM ATP, 0 or 10 nM cGMP, test compound). After 2 h of incubation at RT, the amount of ATP converted to ADP was determined by following the ADP-GLO Max Assay kit instructions. Luminescence signal was read using Envision (PerkinElmer) plate reader. % PKG activation was calculated relative to the fully activated PKG1α using cGMP (100% activation control). The EC_50_ was determined using four-parameter fit analysis of a semi-logarithmic plot of % effect vs. compound concentration.

### Microscale thermophoresis

Microscale thermophoresis (MST) method was used to estimate binding affinities (K_d_) of SMAs for full-length PKG1α (human PKG1α_2–671_) or regulatory domain fragment (bovine PKG1α_79–356_). PKG1α was labeled with NT-647-NHS using the MonolithNT Protein Labeling Kit RED-NHS (NanoTemper, L001) following the manufacturer’s instructions. Briefly, PKG1α was exchanged and diluted into labeling buffer supplemented with 0.01% Triton X-100 and 0.1 mM TCEP to a final concentration of 16.2 μM for full-length human PKG1α_2–671_ and 43 μM for bovine PKG1α_79–356_ fragment. An equal volume of 43.5 μM NT-647-NHS in the same buffer was added to PKG1α and the resulting solution was incubated at room temperature for 30 min. NT-647-labeled PKG1α was purified by gel filtration into assay buffer (50 mM Hepes, pH 7.3, 150 mM NaCl, 10 mM MgCl_2_, 1 mM EDTA, 0.01% BSA, 0.01% Triton X-100, and 2 mM TCEP). The final product had a concentration of 3.1 μM or 4.8 μM and was labeled at 1:1.2 or 1:0.94 stoichiometry of PKG1α:NT-647 for (full-length) human PKG1α_2–671_ and bovine PKG1α_79–356_, respectively. For measurements of compound affinity to the target protein, 200 nl of serially diluted compound in DMSO was dispensed to 10 μl of 10 nM protein containing varying concentrations of cGMP in assay buffer. After a 30 min equilibration period, samples were loaded into Monolith NT.115 hydrophilic capillaries (NanoTemper, K004) and read on a Monolith NT.115 instrument using an MST power of 35% or 60%, for human PKG1α_2–671_ or bovine PKG1α_79–356_ respectively, and a LED power of 90%. The change in normalized fluorescence measured after 5 s of thermophoresis was fit to a four-parameter dose-response equation in GraphPad Prism to determine K_d_ values^23^.

### Fluorescence Polarization (FP) Binding Assays

The modulatory effect of SMAs on cGMP affinity for full-length PKG1α (human PKG1α_2–671_) were tracked using fluorescence polarization method following published procedures^23^. Two assays enabled tracking of changes in cGMP affinity for CNB-A or CNB-B in the presence of SMA.

Briefly, to determine allosteric effects of SMAs on CNB-A, a fixed concentration of the FP probe (8-fluo-cGMP) was used. FP measurements were conducted in 50 mM HEPES, pH 7.3, 150 mM NaCl, 10 mM MgCl2, 1 mM EDTA, 0.01% BSA, 0.01% Triton X-100, and 2 mM TCEP in 384-well plates (Corning 4514) using a final volume of 10 ul. The affinity (K_d_) of the FP probe for CNB-A of PKG1α was determined by mixing 5 ul of a 2x concentration serial dilution of PKG1α with an equal volume of a 2x fixed concentration of the probe. The final concentration of the probe was 0.5 nM ensuring only CNB-A binding was measured. Binding reactions were incubated at room temperature for 1 h and the polarization state of the probe was measured on a PheraStar Plus plate reader (BMG). The affinity of the FP probe was determined by fitting the PKG1α concentration response data in GraphPad Prism using a quadratic equation that accounts for ligand depletion. To determine the effect of SMA on the observed K_d_, the PKG1α serial dilution was repeated in the presence of various SMA concentrations by acoustically dispensing 200 nl of SMA to the PKG1α/probe mixtures before equilibrating for 1 h.

For deducing allosteric effects of SMAs on CNB-B, a fixed-ratio (of PKG1:probe) titration scheme was used. Experimentally, conditions and procedure were the same as described above for determination of affinities at CNB-A with the following exceptions. 10 ul of a fixed-ratio serial dilution of PKG1α:probe (1:2) was added to the assay plate followed by acoustic dispensing of 200 nl of SMA compound in DMSO. Each PKG1α:probe concentration condition was read independently and normalized to a well with a matched probe concentration lacking PKG1α. To determine the probe affinity for CNB-B site from this data, the concentration response curves were fit in GraphPad Prism using a set of previously described equations^23^.

### Protein expression and purification

Residues T204 to F671 were cloned with an N-terminal octahistidine tag followed by a TEV cleavage site (MAHHHHHHHHENLYFQS-_204_TGL.IDF_671_) into pTRIEx4. Protein was expressed in Sf21 insect cells. Cells were seeded into 20 L Sf900II medium (containing 5 μg ml^−1^ Gentamicin) in a 50 L wave bag at 0.65 × 10^6^ cells ml^−1^ and allowed to double in density overnight. The density of the overnight cultures was determined, and cells then infected with P2 BIICS at a multiplicity of infection of 0.2. The infected cultures were incubated for ~72 h at 27 °C. The culture was harvested by centrifugation at 3400 × *g*, 15 min, 4 °C and the pellets were stored at −80 °C.

Cells were lysed in 25 mM Tris/HCl, 500 mM NaCl, 0.5 mM TCEP, 20 mM imidazole, pH 7.0, with protease inhibitor cocktail (Roche Complete) and benzonase added according to manufacturer’s recommendations. The protein was subjected to IMAC using a 5 ml HisTrap crude FF column and a flow rate of 4 ml min^−1^. After a 50 ml wash at 20 mM imidazole the highly purified protein was eluted in a gradient from 20 to 500 mM imidazole over 20 CV. The pooled protein was treated over night with TEV protease (mass ratio 1:30) and then further cleaned up by subtractive IMAC performed essentially as in the first step. A final gel filtration step was conducted using a S200 26/60 pg column equilibrated in 20 mM Tris/HCl pH 8.0, 150 mM NaCl, 1 mM TCEP. Protein was concentrated using regenerated cellulose centrifugal filters with a 10 kDa MWCO to a concentration of 10 mg ml^−1^.

### Crystallization and structure determination

For crystallization, the protein was incubated overnight with 1 mM (ATP, cGMP: 10 mM) of compound (2% DMSO except for nucleotide complexes). Using initial crystals for micro seeding, crystals were readily obtained from multiple positions of the PACT screen (Molecular Dimensions, e.g., wells A05, A03, B06, G08). Crystals grew within a couple of days after mixing 100 nl of protein with 80 nl of reservoir and 20 nl of seed solution. Crystals were harvested by direct flash-cooling in liquid nitrogen without further cryo-preservation treatment or after transfer to 20% ethylene glycol in reservoir.

Diffraction data were collected at the Diamond synchrotron and processed within CCP4 using AIMLESS^43^. A molecular replacement solution was found with PHASER^44^ using chain B of the previously described structure 4KU8^45^ and the kinase domain of PKG 1a (PDB: 6C0T)^26^. The structure was subsequently built and refined with COOT^46^ and autoBUSTER^47^.

### Statistics and reproducibility

Biochemical and biophysical experiments were done in multiple independent trials as indicated in the figure legends (Fig. 2 and Supplementary Figs. 1 and 3). Error estimates were expressed as standard error of the mean (±SE). Error bars in Supplementary Fig. 2 represents SE of the non-linear least squares regression data fit. All statistical analysis were done using GraphPad Prism software.

### Reporting summary

Further information on research design is available in the Nature Portfolio Reporting Summary linked to this article.

## Supplementary information

Supplemental Information

Description of Additional Supplementary Files

Supplementary Data

Reporting Summary

## Data Availability

Raw data underlying graphs in Fig. 2 and Supplementary Information can be found in the included Supplementary Data Excel file. X-ray crystal structures are available at the https://www.rcsb.org via the following ascension codes: 7T4V, 7T4U, 7T4T, 7T4W, and 7SSB. The AlphaFold monomeric model is available as Q13976 https://www.alphafold.ebi.ac.uk. Dimeric models are available on request.
