# Supplementary material for: Structural insights into selective small molecule activation of PKG1α
Source: Commun Biol. 2023 Jul 31;6:798. doi: 10.1038/s42003-023-05095-4 (PMC10390508; doi:10.1038/s42003-023-05095-4)
Supplement: Supplementary file 1 — Supplemental Information [file 42003_2023_5095_MOESM1_ESM.pdf]

Public

# Structural insights into selective small molecule activation of PKG1α

Essam Metwally[1*], Victor Mak[2], Aileen Soriano[3], Matthias Zebisch[4], H. Leonardo Silvestre[4], Paul A. McEwan[4], Grigori Ermakov[5], Maribel Beaumont[5], Paul Tawa[3], John J. Barker[4], Rose Yen[2], Akash Patel[2], Yeon-Hee Lim[2], David Healy[6], Jennifer Hanisak[7], Alan C. Cheng[1], Tom Greshock[2] and Thierry O. Fischmann[8*]

*[1]Modeling and Informatics, MRL, Merck & Co., Inc., 213 E. Grand Avenue, South San Francisco, CA, USA. [2]Discovery Chemistry, MRL, Merck & Co., Inc, 213 E. Grand Avenue, South San Francisco, CA, USA. [3]Quantitative Biosciences, MRL, Merck & Co., Inc., Kenilworth, NJ, USA. [4]Evotec (UK) Ltd, 114 Innovation Drive, Milton Park, Abingdon, Oxfordshire OX14 4RZ, United Kingdom. [5]Discovery Bioanalytics, MRL, Merck & Co., Inc, 213 E. Grand Avenue, South San Francisco, CA, USA. [6]Discovery Biology, MRL, Merck & Co., Inc, Boston, MA, USA. [7]Discovery Chemistry, MRL, Merck & Co., Inc., Kenilworth, NJ, USA. [8]Protein and Structural Chemistry, MRL, Merck & Co., Inc., Kenilworth, NJ, USA.*

** Corresponding authors:*

*Essam Metwally; E-mail: essam.metwally@merck.com*

*Thierry Fischmann; E-mail: thierry.fischmann@merck.com*

Public

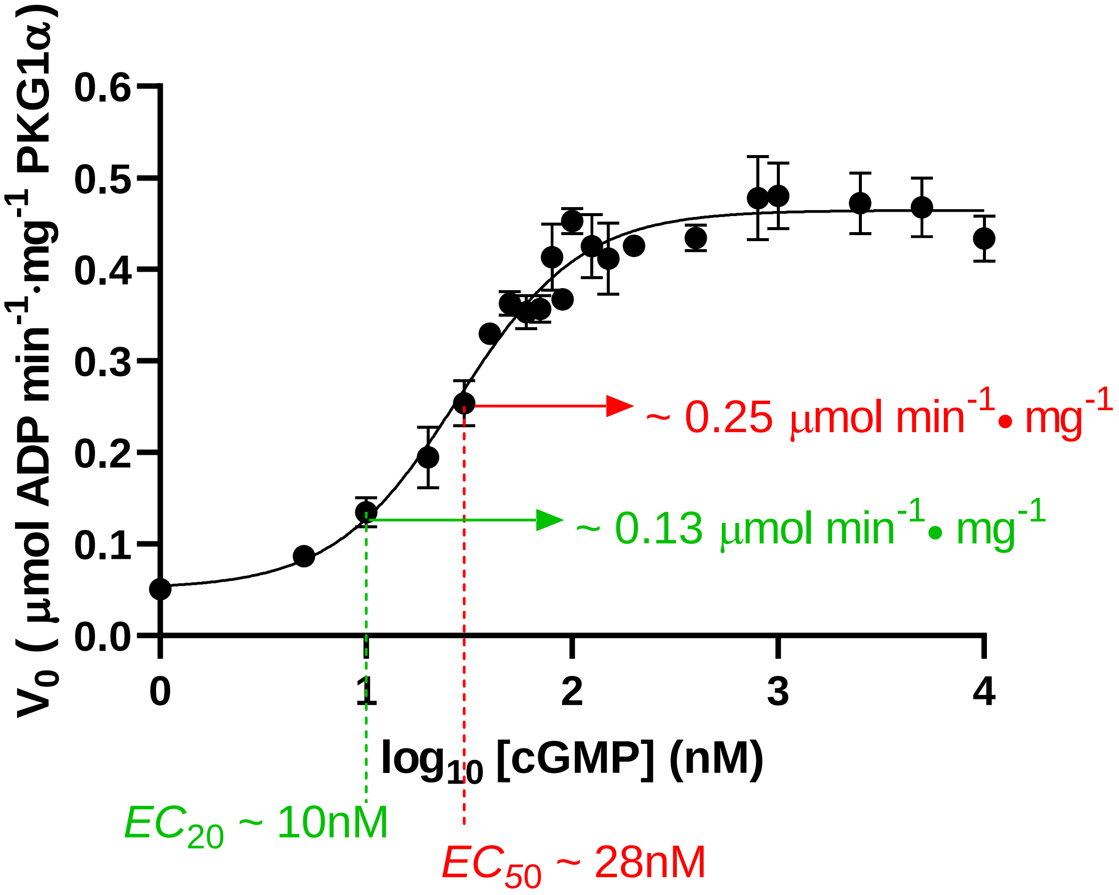


*Supplementary Figure 1 cGMP activation of full length PKG1α. Initial velocities were derived by following production of ADP with time using ADP-Glo kinase assay. cGMP $EC_{20}$ and $EC_{50}$ values were estimated from 4-parameter non-linear fit of concentration-response data using PRISM software. Error bars indicate standard error (SE) from 4 independent trials.*

Public

a)

| | [cGMP] (nM) | $K_D$ (µM) | | | | |
|---|---|---|---|---|---|---|
| | | SMA1 | SMA2 | SMA3 | SMA4 | SMA5 |
| ○ | 0 | weak | weak | weak | weak | weak |
| ● (grey) | 10 | * | 9.7 | 1.0 | 0.52 | * |
| ● | 100 | 1.1 | 0.61 | 0.16 | 0.14 | 0.47 |

* SMA binding detected; incomplete binding profile due to limited solubility

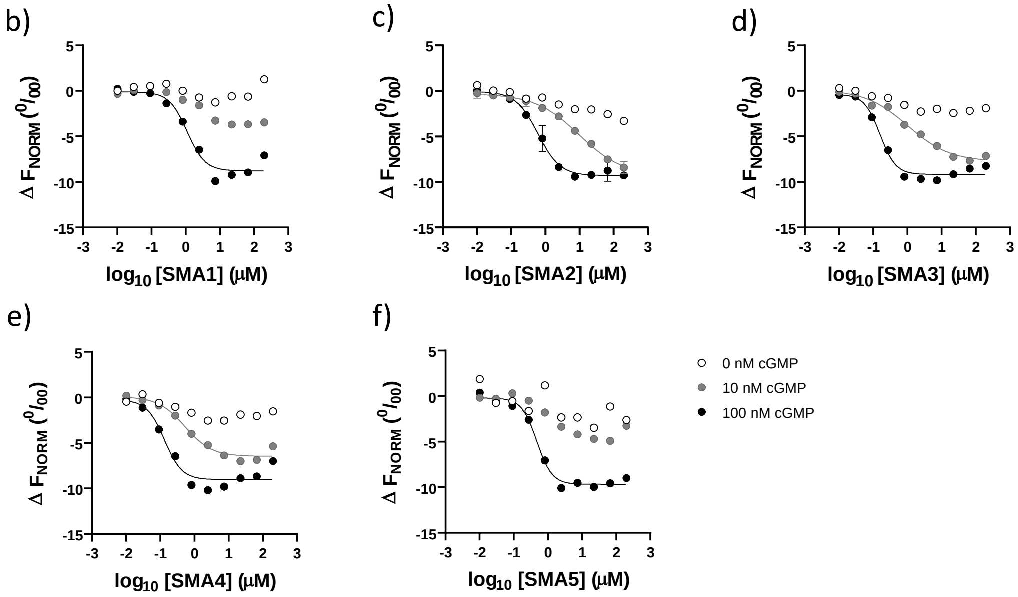


*Supplementary Figure 2 Increase in binding affinity of SMAs for PKG1α in the presence of cGMP. a) Table shows increasing binding affinity (Kd) of SMAs for full length PKG1a at increasing concentration of cGMP. $K_d$ values were estimated using Microscale Thermophoresis (MST): changes in thermophoresis of PKG1α upon binding of SMA1 (b), SMA2 (c), SMA3 (d), SM4 (e) and SMA5 (f) were tracked at the indicated (fixed) concentration of cGMP. Error bars represent SE of nonlinear least squares regression. Increase in binding affinity of SMAs for PKG1α in the presence of cGMP.*

Public

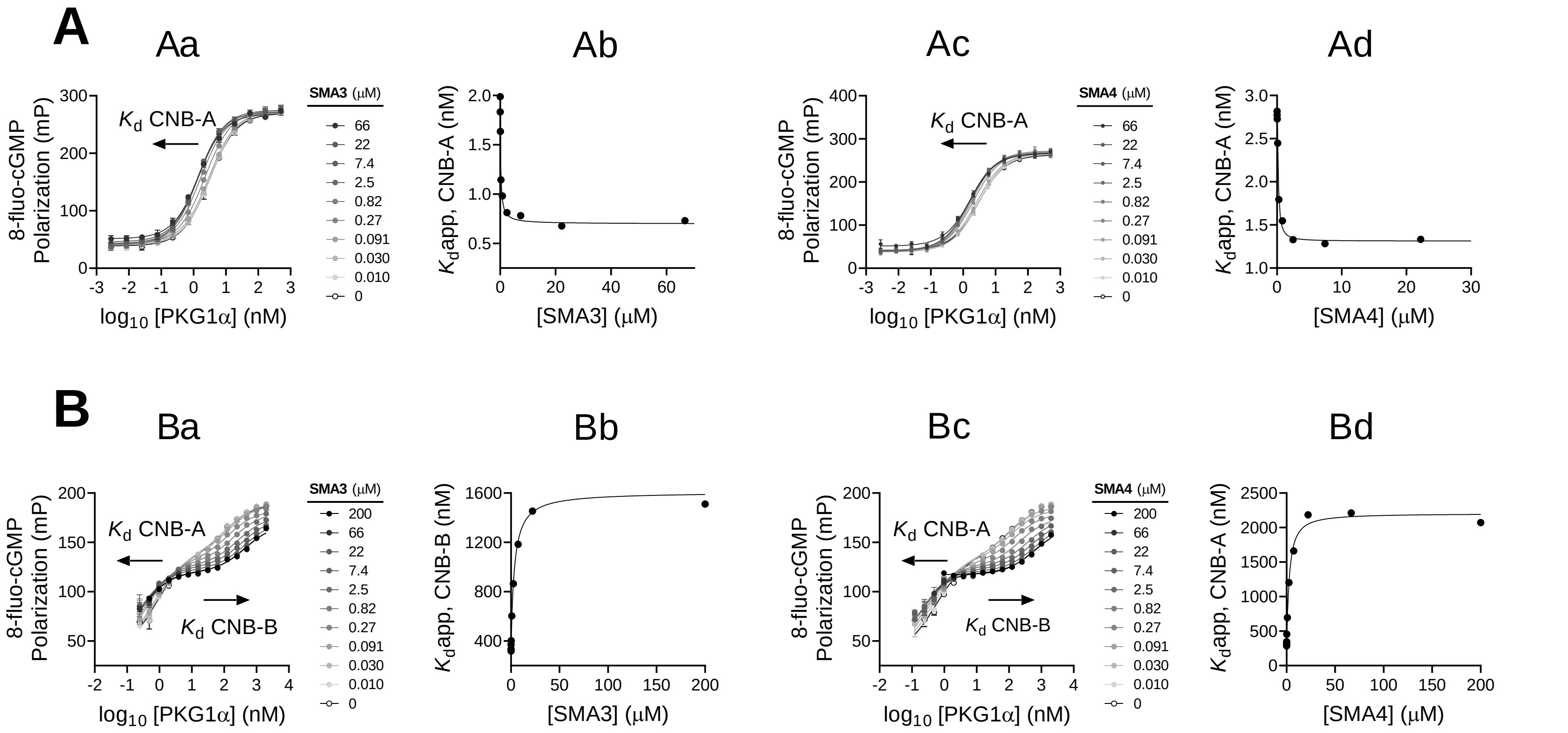


*Supplementary Figure 3 Fluorescence polarization assays measuring effect of SMAs on cGMP binding to CNB-A and CNB-B in full-length PKG1α (PKG1α $_{2\text{-}671}$). A, enhancement of cGMP binding affinity for CNB-A at increasing concentrations of SMAs. The increase in apparent affinity of 8-fluo-cGMP for CNB-A site with increasing concentration of SMA3 (Aa) or SMA4 (Ac) is observed in the left shift trend of the concentration response curves. Error bars represent SE from two replicate measurements. The curvilinear relationship between affinity of 8-fluo-cGMP for CNB-A and concentration of SMA3 (Ab) or SMA4 (Ad) indicate SMAs bind to a site allosteric to CNB-A ($K_d$ values are from best fit curves from panel Aa and Ac, respectively). B, negative modulation of cGMP affinity for CNB-B. Bb, Sequential two-site equilibrium binding of 8-fluocGMP to PKG1α. A decrease in apparent affinity of 8-fluo-cGMP for CNB-B with increasing concentration of SMA3 (Ba) or SMA4 (Bc) is observed as a right shift in the portion of the concentration–response curve corresponding to binding to CNB-B. Error bars represent SE from two replicate measurements. Curvilinear relationship between apparent 8-fluo-cGMP $K_d$ and concentration of SMA3 (Bb) or SMA4 (Bd) indicating piperidines bind to a site allosteric to CNB-B.*

Public

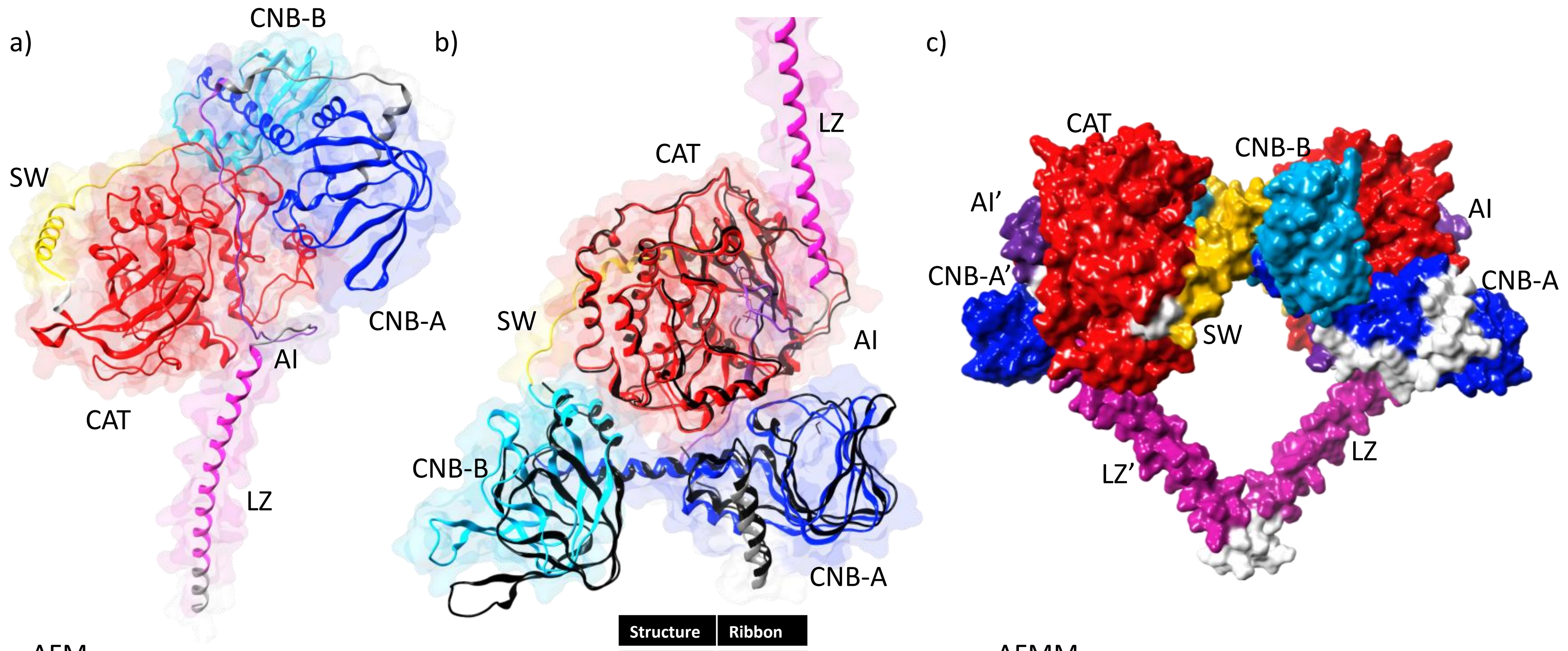


| Structure | Ribbon |
|---|---|
| AFM | Colored |
| 7LV3 | Black |

*Supplementary Figure 4.* ***AlphaFold predicted structures of the PKG1α monomer and dimer as compared to 7LV3.*** *a) AlphaFold predicted monomeric PKG1α structure (AFM). Note that the AI domain is packed into CAT as suggested by the 7LV3. b) Overlay of 7LV3 swapped monomer (black ribbon), where the co-located domains are treated as a monomer, with AFM. The ribbon traces are quite similar with deviations being due to positioning of inter-domain helices. c) AlphaFold-Multimer predicted structure (AFMM) predicts an extended state where the catalytic domain is co-located with the regulatory domain of the opposite chain. The AI sequences are predicted to inhibit the trans-chains. Despite being able to recapitulate 7LV3, it was unable to make predictions with respect to the LZ and its dimerization.*
