# Supplementary material for: Structural insights into selective small molecule activation of PKG1α
Source: Commun Biol. 2023 Jul 31;6:798. doi: 10.1038/s42003-023-05095-4 (PMC10390508; doi:10.1038/s42003-023-05095-4)
Supplement: Supplementary file 2 — Description of Additional Supplementary Files [file 42003_2023_5095_MOESM2_ESM.pdf]

Description of Additional Supplementary Files

File name: Supplementary Data
Description: Source data behind all graphs in the manuscript (Figure 2, and Supplementary Figures 1-3).
